# Exploring factors associated with self‐rated health in individuals with diabetes and its impact on quality of life: Evidence from the Survey of Health, Ageing, and Retirement in Europe

**DOI:** 10.1111/1753-0407.13522

**Published:** 2024-01-02

**Authors:** Rosa Marie Brückner, Aline Schönenberg, Rebecca Wientzek, Mandy Schreiber, Tino Prell

**Affiliations:** ^1^ Department of Geriatrics Halle University Hospital Halle Germany; ^2^ Department of Internal Medicine II Halle University Hospital Halle Germany; ^3^ Department of Neurology Jena University Hospital Jena Germany

**Keywords:** diabetes, older adults, quality of life, self‐rated health

## Abstract

**Background:**

Self‐rated health (SRH), a measure of self‐reported general health, is a robust predictor of morbidity and mortality in various populations, including people with diabetes. Diabetes is negatively associated with SRH and quality of life (QoL). Little is known about how people with diabetes rate their health and which aspects influence the rating. Also, the predictive value of SRH on future QoL has not yet been evaluated.

**Methods:**

We analyzed data from 46 592 participants of the Survey of Health, Ageing and Retirement in Europe (SHARE). Using linear regression, we aimed to determine which sociodemographic, socioeconomic, medical, social, mental, and health behavior factors determine SRH in people with diabetes. In addition, we analyzed the predictive value of SRH on future QoL using the generalized estimating equations procedure.

**Results:**

We determined that country, current job situation, hospitalization, pain, polypharmacy, memory, eyesight, activities of daily living, number of chronic diseases, and depression are all linked to SRH. Together these variables explained 38% of the SRH's variance, whereas depression, pain, and memory had the greatest influence on SRH of people with diabetes. We also found that SRH independently predicted future QoL, supported by a regression coefficient of *β* = −1.261 (Wald chi‐square test, *χ* ^*2*^ = 22.097, *df* = 1, *p* < .05).

**Conclusions:**

As SRH is linked to future QoL, we conclude that incorporating SRH assessment into medical evaluations can help health care professionals gaining a more comprehensive understanding of an individual's health trajectory and supporting patients to enhance their QoL.

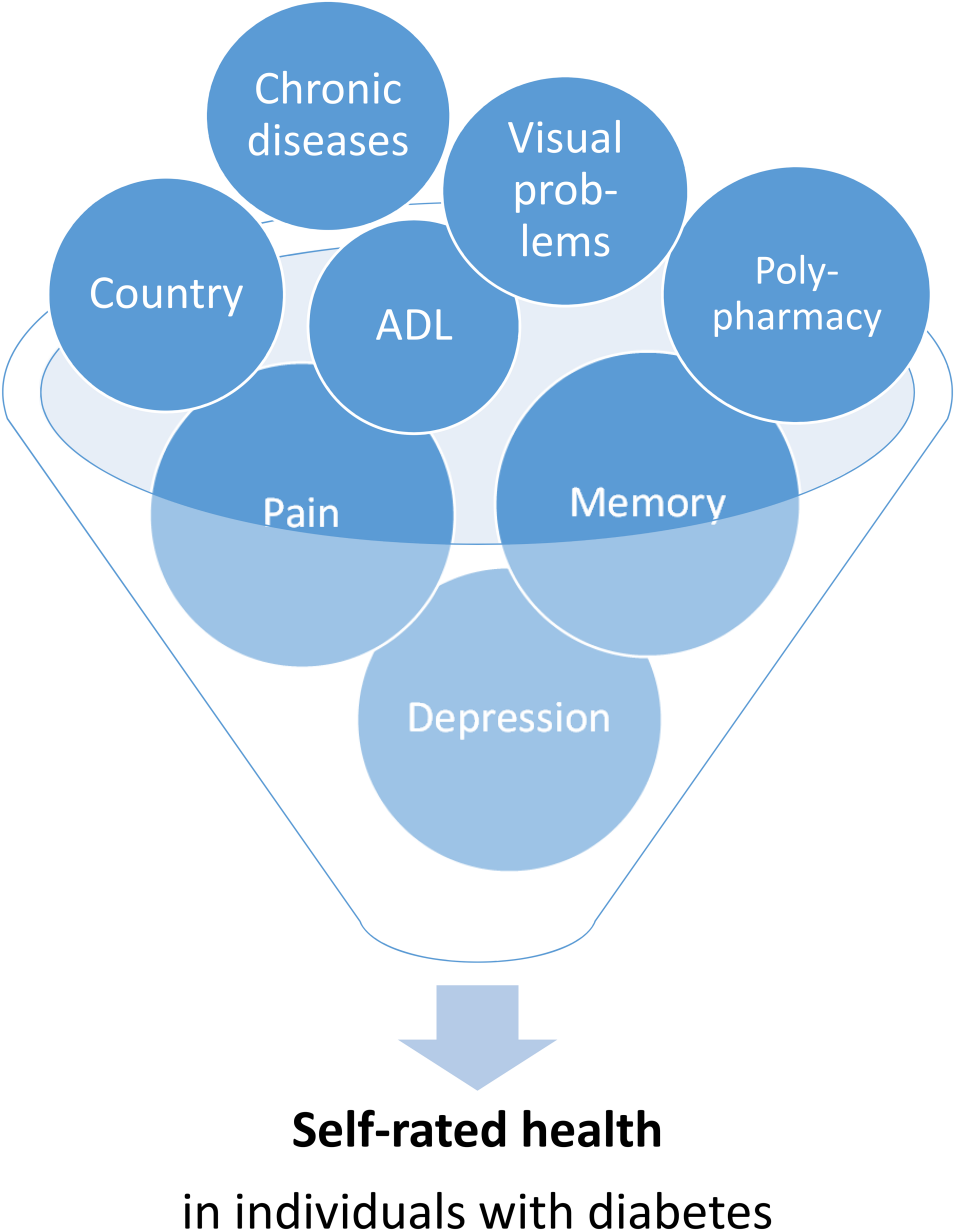

## Graphical abstract

Highlights

- Individuals with diabetes tend to report their health as less than very good more frequently compared to individuals without diabetes
- We have identified several factors that influence self‐rated health in individuals with diabetes. Depression, pain, and memory function emerged as the most influential factors.
- Self‐rated health was determined to be an independent predictor of quality of life.

## 1 INTRODUCTION

Self‐rated health (SRH) is a relevant indicator of an individual's overall health status and well‐being. It reflects the respondent's subjective assessment of their physical and mental health status and can be used to predict many health behaviors and outcomes.^1^, ^2^, ^3^, ^4^, ^5^ Previous research has found an association between poor SRH and a higher risks of chronic diseases, such as diabetes.^6^, ^7^, ^8^, ^9^, ^10^, ^11^

SRH can be measured with a single item and therefore has the ability to efficiently summarize the combination of different risk factors that influence health. As such, SRH is a simple and economical way of measuring health. The wording of the items used to measure SRH, for example “Would you say your health is⋯”, rated on a five‐point Likert scale ranging from “excellent” to “poor,”^12^, ^13^, ^14^, ^15^ is broad, leaving respondents free to decide on which aspects to base their health rating. According to Jylhä,^16^ the rating is a subjective summary of the health information available to the respondent. These include medical diagnoses, functional status observations, and physical sensations. Additionally, factors like strengths and risks that may affect the future health, formal signs of illness such as disability pensions or prescribed medications, personal expectations and experiences, reference groups, and individual characteristics may influence SRH. Finally, sociodemographic and cultural factors may also cause differences in SRH. This may be due to real differences in health status, different levels of health knowledge, or linguistic factors resulting from language translation.^16^ With different health conditions, the individual meaning of SRH can vary significantly. Those without chronic conditions may see SRH as an indicator of their general well‐being, whereas those with conditions such as diabetes may see it as a reflection of the problems associated with their illness.^17^

To understand what SRH really measures, previous studies have attempted to identify determinants and measure their impact on the rating of SRH. For example, Lazarevic and Brandt^18^ identified functioning, diseases, and pain as the main determinants explaining the variance in SRH. However, as particular conditions may influence the way people rate their health, there is still a limited understanding of the determinants of SRH in people with chronic diseases, such as diabetes.

Diabetes is one of the world's most common diseases. More than half a billion people worldwide are living with diabetes, representing 10.5% of the adult population. A significant increase in the prevalence is predicted for the future.^19^ Diabetes can affect an individual's physical, mental, and social health^20^, ^21^, ^22^ and is associated with reduced quality of life (QoL).^23^, ^24^ SRH is associated with lifestyle factors such as diet, exercise, alcohol consumption, and obesity, all of which are also linked to diabetes.^25^, ^26^ Higher mortality, poorer glycemic control, and higher rates of complications and hospitalizations are found in people with diabetes who assess their health as moderate or poor compared to individuals with good or excellent SRH.^11^, ^27^, ^28^, ^29^ Additionally, depression and disability in people with diabetes are associated with poor SRH.^17^

Until now, little is known about how SRH is composed in people with diabetes. Previous studies have focused on the relationship between SRH and, for example, physical health or sociodemographic factors or the correlation between SRH and a variety of sociodemographic factors.^30^, ^31^ However, it is important to understand the relative importance of physiological and psychosocial factors that influence people's SRH. It is therefore necessary to analyze a large dataset that includes not only disease‐specific but also psychosocial and medical variables.

The concept of QoL embodies the holistic approach to health advocated by the World Health Organization.^32^ In older adults, QoL has been found to be associated with several factors that are also related to SRH, such as social network, physical activity, memory, depression, activities of daily living (ADL), and chronic diseases such as diabetes.^33^, ^34^, ^35^ This raises the question whether SRH is predictive for QoL in older adults with diabetes.

This study aims to address two key issues: First, we want to determine the extent to which a comprehensive set of sociodemographic, socioeconomic, medical, social, mental, health care utilization, and health behavior indicators can explain the variance of SRH in people with diabetes. Understanding how people with diabetes rate their health and what information they rely on can help clinicians to better understand their patients' needs and develop appropriate interventions to support healthy living with diabetes.

Second, this study aims to determine the predictive value of SRH for future QoL in people with diabetes, as the construct QoL is considered to be an important correlate of diabetes^23^, ^24^ and therefore needs more awareness and monitoring.

To address these two key issues, we used data from a large European cohort study, as SRH levels vary across different European countries due to different cultural and socioeconomic factors.^36^, ^37^, ^38^

## 2 METHODS

### 2.1 Origin of data

All data used in this paper come from the Survey of Health, Ageing and Retirement in Europe (SHARE). SHARE is a longitudinal research infrastructure that collects comprehensive data on health, social, environmental, and economic factors among older adults across several European countries. The target population are individuals aged 50 years and over. The study is carried out in 28 countries and is designed as a computer‐assisted personal interviewing. It consists of several waves, with data collection taking place at regular intervals. In this paper we use data from SHARE waves 7 and 8.

### 2.2 Dependent variables

In SHARE wave 8, SRH was operationalized with a single item, based on the questionnaire Short Form 36 by Ware and Gandek^39^: “Would you say your health is⋯” with the option to choose from a five‐point Likert scale, with response options of “Excellent,” “Very good,” “Good,” “Fair,” and “Poor.”^40^

To measure QoL in older adults independently of its determinants, such as health status, material circumstances, and social network, Higgs and colleagues developed the Control, Autonomy, Self‐Realization and Pleasure‐19 (CASP‐19) scale, based on Maslow's^41^ theory of human needs. The authors suggested that QoL “should be assessed as the degree that human needs are satisfied.”^42^ It includes the dimensions of control, autonomy, self‐realization, and pleasure. In SHARE, QoL is operationalized with an adapted 12‐item version of the scale (CASP‐12).^40^

### 2.3 Independent variables

Table 1 provides an overview of independent variables included in this study that are all known to be associated with SRH according to existing literature. These variables include common sociodemographic factors such as age,^47^ sex,^48^, ^49^ marital status, education level, socioeconomic status,^2^, ^50^, ^51^, ^52^ number of limitations in daily living,^53^ body mass index (BMI),^54^ cognitive function,^55^ depression,^56^ physical activity,^57^, ^58^ hand grip strength,^55^ sensory impairments,^55^ pain,^59^ previous hospitalization,^60^ social network satisfaction, social connectedness,^61^, ^62^, ^63^ and health behaviors, such as drinking alcohol and smoking.^47^, ^56^, ^64^, ^65^ We have also included the country of survey, recognizing that there may be systematic differences in responses between and within countries due to sociocultural differences. Furthermore, countries with a higher proportion of elderly individuals are expected to have fewer individuals reporting good health. A detailed description of the operationalization and measurement of the variables can be found in the scales manual of SHARE.^40^

**TABLE 1:** Independent variables.

Independent variable		Level of measurement
**Sociodemographics**		
Age		Metric
Sex		Nominal
Marital status		Nominal
Country		Nominal
**Socioeconomic**		
Education	Years of education	Metric
Health literacy	How often help needed for reading documents from doctor/pharmacy	Metric
Net worth	Household net worth	Metric
Employment status	Current job situation	Nominal
**Medical factors**		
BMI	Body mass index	Metric
Chronic diseases	Number of chronic diseases	Metric
Polypharmacy	At least taking 5 different drugs a typical day	Nominal
Physical inactivity		Nominal
Grip strength	Max. of grip strength measure	Metrisch
Eyesight	Eyesight reading	Nominal
Hearing		Nominal
Pain	Troubled with pain	Nominal
Adl	Number of limitations with activities of daily living	Metric
**Social factors**		
Sn size	Social network size	Metric
Sn satisfaction	Social network satisfaction	Metric
Sn scale	Scale of social connectedness^43^	Metric
Loneliness	Three‐Item Loneliness Scale^44^	Metric
**Mental factors**		
Depression	Depression scale EURO‐D^45^	Metric
Fluency	Score of verbal fluency test^46^	Metric
Memory	Memory (self‐rated)	Nominal
Sleep	Trouble sleeping	Nominal
**Health care utilization**		
Hospital	In hospital during last 12 months	Nominal
Nursing home	In a nursing home during last 12 months	Nominal
**Health behavior**		
Smoke	Smoke at the present time	Nominal
Alcohol	How often six or more drinks the last 3 months	Nominal

### 2.4 Statistical analyses

Descriptive statistics were first conducted to characterize the sample. Univariate group comparisons were performed using Student's *t* test, *U*‐test, or chi‐square test as necessary. We performed a multiple linear regression analysis using backward selection to determine the optimal model for explaining variance in SRH and to assess the degree to which independent variables account for the variance in SRH (SRH was treated as dependent variable). We assumed that SRH is a continuous concept of subjective health and that a dichotomization of the variable would lead to a significant loss of information. Thus, we treated SRH as a continuous variable. To account for potential country influences and avoid complications arising from dummy coding of 23 countries, we categorized these countries into seven distinct groups based on their standardized regression coefficients (beta). Each group was assigned a value of “1” to indicate membership and “0” otherwise. An overview of the categorization is provided in Table S1. Since linearity was not found for the variables *net worth* and *bmi*, we eliminated them from the analysis. Multicollinearity was found for the variables *scale of social connectedness* and *social network size* (*r* = 0.849) as well as for the dummy variables *retired* and *employed or self‐employed* (*r* = −0.763). As a result, *social network size* and *retired* were omitted from the analyses. Outliers were not removed since the maximum leverage point was <0.2.^66^ The model showed no autocorrelation, with a Durbin‐Watson statistic of 1.984. All other requirements have been approved (Gaussian distribution of residuals and homoscedasticity).

We analyzed the predictive value of SRH on QoL by selecting participants who had a history of diabetes or high blood sugar or were currently diagnosed with the condition and who had likewise taken part in SHARE Wave 7. We used generalized estimating equations (GEE) to account for possible correlations between independent variables from repeated measures at waves 7 and 8. We thereby treated QoL as a dependent variable. The predictors used for performing the GEE were the variables that accounted for the variance in SRH in our initial analyses and were subsequently included in the model.

All statistical analyses were conducted using IBM SPSS Statistics (Version 25). The statistical significance was determined with *p* < .05. In cases of missing data, pairwise deletion was utilized.

## 3 RESULTS

### 3.1 Study sample

We analyzed data from 46.592 individuals across 27 countries (26 European countries and Israel) who participated in the eighth wave of the European cohort study SHARE.^12^, ^13^, ^14^ The study involved 42.5% male and 57.5% female participants, with a mean age of 70.2 years (SD = 9.4) and an age range of 32 to 103 years.

Of the study's participants, 14.9% reported having a history of diabetes or high blood sugar or were currently diagnosed with the condition. The mean age of these individuals was 72.4 years, and the mean age at which they were diagnosed with diabetes was 56.8 years (SD = 12.3). 47.7% of those with a history of diabetes or high blood sugar were male and 52.3% female. It is worth noting that 3031 of these participants also participated in SHARE Wave 7^67^ and were subsequently included in further analyses.

### 3.2 Group comparisons

Supplement Table S2 displays the frequencies and percentages of responses in each variable for participants with and without diabetes, as well as the *p* values and effect sizes. Most variables showed significant impacts of diabetes, except for *nursing home* (Having been in a nursing home during last 12 months) and *sn satisfaction* (social network satisfaction). Individuals with diabetes reported a higher incidence of a SRH “less than very good” (92.2%) compared to individuals without diabetes (75.6%) (*p* < .001, *V* = 0.143).

Table 2 presents an overview of SRH in relation to different variables among individuals with diabetes. Those with lower SRH reported weaker social connectedness, poorer education, lower health literacy, increased limitations with ADL, decreased hand grip strength, poorer cognitive performance, and more depressive symptoms when compared to individuals with good or excellent SRH. The highest effect sizes were found for depression, verbal fluency, and hand grip strength.

**TABLE 2:** Comparison between people with diabetes with good/excellent SRH and less than very good SRH (Wave 8).

	Very good/excellent (N = 537)	Less than very good (N = 6384)	Total (N = 6921)	*p* value	Cohen's D or Cramer's V
**SRH**				<.001	−2.815
Mean (SD)	1.8 (0.4)	3.8 (0.7)	3.7 (0.9)		
**Age (years)**				<.001	−0,261
Mean (SD)	70.3 (8.4)	72.5 (8.6)	72.4 (8.6)		
**Sex**				<.001	0.058
Male	310 (57.7%)	2994 (46.9%)	3304 (47.7%)		
Female	227 (42.3%)	3390 (53.1%)	3617 (52.3%)		
**Marital status**				<.001	0.049
Married, living with spouse	371 (69.1%)	3999 (62.6%)	4370 (63.1%)		
Registered partnership	6 (1.1%)	60 (0.9%)	66 (1.0%)		
Married, not living with spouse	5 (0.9%)	76 (1.2%)	81 (1.2%)		
Never married	19 (3.5%)	316 (4.9%)	335 (4.8%)		
Divorced	46 (8.6%)	445 (7.0%)	491 (7.1%)		
Widowed	90 (16.8%)	1488 (23.3%)	1578 (22.8%)		
**Country**				<.001	0.199
Austria	17 (3.2%)	209 (3.3%)	226 (3.3%)		
Germany	30 (5.6%)	425 (6.7%)	455 (6.6%)		
Sweden	52 (9.7%)	231 (3.6%)	283 (4.1%)		
Netherlands	14 (2.6%)	197 (3.1%)	211 (3.0%)		
Spain	32 (6.0%)	371 (5.8%)	403 (5.8%)		
Italy	13 (2.4%)	282 (4.4%)	295 (4.3%)		
France	16 (3.0%)	295 (4.6%)	311 (4.5%)		
Denmark	54 (10.1%)	140 (2.2%)	194 (2.8%)		
Greece	45 (8.4%)	439 (6.9%)	484 (7.0%)		
Switzerland	32 (6.0%)	137 (2.1%)	169 (2.4%)		
Belgium	35 (6.5%)	217 (3.4%)	252 (3.6%)		
Israel	18 (3.4%)	226 (3.5%)	244 (3.5%)		
Czech Republic	36 (6.7%)	544 (8.5%)	580 (8.4%)		
Poland	11 (2.0%)	412 (6.5%)	423 (6.1%)		
Luxembourg	9 (1.7%)	112 (1.8%)	121 (1.7%)		
Hungary	10 (1.9%)	172 (2.7%)	182 (2.6%)		
Slovenia	34 (6.3%)	376 (5.9%)	410 (5.9%)		
Estonia	6 (1.1%)	425 (6.7%)	431 (6.2%)		
Croatia	14 (2.6%)	166 (2.6%)	180 (2.6%)		
Lithuania	3 (0.6%)	145 (2.3%)	148 (2.1%)		
Bulgaria	8 (1.5%)	123 (1.9%)	131 (1.9%)		
Cyprus	12 (2.2%)	107 (1.7%)	119 (1.7%)		
Finland	13 (2.4%)	163 (2.6%)	176 (2.5%)		
Latvia	0 (0.0%)	76 (1.2%)	76 (1.1%)		
Malta	16 (3.0%)	145 (2.3%)	161 (2.3%)		
Romania	4 (0.7%)	162 (2.5%)	166 (2.4%)		
Slovakia	3 (0.6%)	87 (1.4%)	90 (1.3%)		
**Education**				<.001	0.325
Mean (SD)	11.7 (4.4)	10.4 (4)	10.5 (4.1)		
**Health literacy**				<.001	0.099
Always	23 (4.3%)	649 (10.2%)	672 (9.8%)		
Often	8 (1.5%)	362 (5.7%)	370 (5.4%)		
Sometimes	39 (7.3%)	673 (10.6%)	712 (10.3%)		
Rarely	44 (8.2%)	754 (11.9%)	798 (11.6%)		
Never	423 (78.8%)	3917 (61.6%)	4340 (63%)		
**Net worth**				<.001	0.343
Mean (SD)	359455.1 (623089)	194247.1 (467622.6)	207065.6 (483453.5)		
**Employment status**				<.001	0.088
Retired	404 (76.2%)	4883 (77.7%)	5287 (77.6%)		
Employed or self‐employed (including working for family business)	81 (15.3%)	497 (7.9%)	578 (8.5%)		
Unemployed	3 (0.6%)	90 (1.4%)	93 (1.4%)		
Permanently sick or disabled	3 (0.6%)	264 (4.2%)	267 (3.9%)		
Homemaker	34 (6.4%)	455 (7.2%)	489 (7.2%)		
Other	5 (0.9%)	95 (1.5%)	100 (1.5%)		
**BMI**				<.001	0.297
Mean (SD)	27.9 (4.3)	29.5 (5.3)	29.3 (5.3)		
**Chronic diseases**				<.001	−0.609
Mean (SD)	2.5 (1.3)	3.6 (1.7)	3.5 (1.7)		
**Polypharmacy**				<.001	0.158
Yes	163 (31%)	3806 (60.2%)	3969 (58%)		
No	363 (69%)	2513 (39.8%)	2876 (42%)		
**Physical inactivity**				<.001	
No	508 (94.6%)	4934 (77.3%)	5442 (78.6%)		
Yes	29 (5.4%)	1450 (22.7%)	1479 (21.4%)		
**Grip strength**				<.001	0.433
Mean (SD)	35.1 (11.4)	30.3 (11)	30.7 (11.1)		
**Eyesight**				<.001	0.209
Excellent	159 (29.6%)	638 (10.0%)	797 (11.5%)		
Very good	205 (38.2%)	1497 (23.4%)	1702 (24.6%)		
Good	125 (23.3%)	2729 (42.7%)	2854 (41.2%)		
Fair	35 (6.5%)	1016 (15.9%)	1051 (15.2%)		
Poor	13 (2.4%)	504 (7.9%)	517 (7.5%)		
**Hearing**				<.001	0.162
Excellent	105 (19.6%)	565 (8.9%)	670 (9.7%)		
Very good	201 (37.4%)	1314 (20.6%)	1515 (21.9%)		
Good	157 (29.2%)	2821 (44.2%)	2978 (43%)		
Fair	64 (11.9%)	1324 (20.7%)	1388 (20.1%)		
Poor	10 (1.9%)	360 (5.6%)	370 (5.3%)		
**Pain**				<.001	0.175
Yes	132 (24.6%)	3639 (57.1%)	3771 (54.6%)		
No	405 (75.4%)	2730 (42.9%)	3135 (45.4%)		
**ADL**				<.001	−0.343
Mean (SD)	0.1 (0.5)	0.5 (1.3)	0.5 (1.2)		
SN size				<.001	0.182
Mean (SD)	2.8 (1.7)	2.5 (1.6)	2.5 (1.6)		
**SN satisfaction**				<.001	0.242
Mean (SD)	9.2 (1.1)	8.9 (1.5)	8.9 (1.5)		
**SN scale**				<.001	0.179
Mean (SD)	2 (1)	1.8 (1)	1.8 (1)		
**Loneliness**				<.001	−0.356
Mean (SD)	3.7 (1.2)	4.2 (1.6)	4.2 (1.6)		
**Depression**				<.001	−0.640
Mean (SD)	1.5 (1.7)	3 (2.5)	2.9 (2.4)		
**Fluency**				<.001	0.411
Mean (SD)	21.1 (7.3)	18.1 (7.3)	18.3 (7.3)		
**Memory**				<.001	0.210
Excellent	65 (12.1%)	275 (4.3%)	340 (4.9%)		
Very good	193 (35.9%)	891 (14%)	1084 (15.7%)		
Good	217 (40.4%)	2945 (46.1%)	3162 (45.7%)		
Fair	56 (10.4%)	1809 (28.3%)	1865 (26.9%)		
Poor	6 (1.1%)	464 (7.3%)	470 (6.8%)		
**Sleep**				<.001	0.116
Trouble with sleep or recent change in pattern	117 (21.8%)	2666 (43%)	2783 (41.3%)		
No trouble sleeping	419 (78.2%)	3541 (57%)	3960 (58.7%)		
**Hospital**				<.001	0.083
No	483 (89.9%)	4928 (77.2%)	5411 (78.2%)		
Yes	54 (10.1%)	1456 (22.8%)	1510 (21.8%)		
**Nursing home**				.5342	‐
Yes, temporarily	1 (0.2%)	29 (0.5%)	30 (0.4%)		
Yes, permanently	0 (0%)	5 (0.1%)	5 (0.1%)		
No	529 (99.8%)	6245 (99.5%)	6774 (99.5%)		
**Smoke**				.4622	‐
Yes	63 (26.9%)	781 (29.2%)	844 (29.0%)		
No	171 (73.1%)	1894 (70.8%)	2065 (71.0%)		
**Alcohol**				<.001	0.096
Daily or almost daily	8 (1.5%)	76 (1.2%)	84 (1.2%)		
Five or six days a week	8 (1.5%)	21 (0.3%)	29 (0.4%)		
Three or four days a week	5 (0.9%)	58 (0.9%)	63 (0.9%)		
Once or twice a week	18 (3.4%)	152 (2.4%)	170 (2.5%)		
Once or twice a month	32 (6.0%)	240 (3.8%)	272 (3.9%)		
Less than once a month	70 (13.0%)	403 (6.3%)	473 (6.8%)		
Not at all in the last 3 months	396 (73.7%)	5425 (85.1%)	5821 (84.2%)		

*Note*: *p* value of Student's *t* or chi‐square test. ADL, activities of daily living; BMI, body mass index; SRH, self‐rated health.

### 3.3 Linear model

The final overall model had an *R* ^*2*^ of 0.40 (adjusted *R* ^*2*^ = 0.38), which in accordance with Cohen's (1988) standards indicated a high goodness of fit. The variables *memory*, *adl*, *chronic diseases*, *eyesight*, and *depression* remained in the model as well as the dummy variables *country_group1*, *country_group2*, *country_group3*, *employment status* = *permanently sick or disabled*, *hospital = Yes*, *pain = No*, *physical inactivity* = *Yes*, *polypharmacy* = *No*. They significantly predicted SRH with *F* (13, 473) = 24.19, *p* < .001.

Table 3 displays the standardized and nonstandardized regression coefficients of the variables in the model. It is thereby important to note the polarity of the dependent variable: A higher score on the SRH variable indicates a poorer SRH. According to the standardized betas, *depression*, *pain* and *memory* are the variables that had the greatest impact on SRH.

**TABLE 3:** Factors associated with self‐rated health (SRH) in people with diabetes.

	Coefficient	SE	Standardized beta	*T*	Sig.	95% confidence interval for Beta	
						Lower limit	Upper limit
(constant)	2.593	0.161		16.060	<0.001	2.276	2.910
Chronic diseases	0.060	0.022	0.116	2.733	0.007	0.017	0.104
Eyesight	0.106	0.032	0.127	3.298	0.001	0.043	0.170
ADL	0.072	0.030	0.100	2.376	0.018	0.012	0.131
Depression	0.061	0.015	0.167	3.976	<0.001	0.031	0.091
Memory	0.124	0.037	0.130	3.374	<0.001	0.052	0.196
Employment status	0.318	0.165	0.070	1.925	0.055	−0.007	0.643
Physical inactivity	0.257	0.088	0.119	2.925	0.004	0.084	0.429
Polypharmacy	−0.179	0.072	−0.100	−2.496	0.013	−0.320	−0.038
Pain	−0.251	0.070	−0.141	−3.604	<0.001	−0.388	−0.114
Hospital	0.153	0.079	0.071	1.922	0.055	−0.003	0.309
Country_group1	0.346	0.140	0.090	2.469	0.014	0.071	0.622
Country_group2	0.231	0.086	0.098	2.696	0.007	0.063	0.399
Country_group3	0.201	0.122	0.060	1.651	0.099	−0.038	0.440

*Note*: Variables initially included in the model: *age*, *education* (dn041), *health literacy* (hc889_), *age diabetes* (ph009_5), *chronic diseases* (chronicw8c), *grip strength* (maxgrip), *eyesight* (ph044_), *hearing (ph046_)*, *adl*, *sn_satisfaction*, *sn_scale*, *loneliness*, *depression* (eurod), *fluency* (cf010_), *memory* (cf103_), *marital status* (dn014_; dummy variables), *employment status* (ep005_; dummy variables), *nursing home* (hc029_; dummy variables), *sex* (dn042;_dummy variables), *physical inactivity* (phactiv; dummy variables), polypharmacy (ph082_; dummy variables), *pain* (ph084_; dummy variables), *hospital* (hc012_; dummy variables), *smoke* (br002_; dummy variables), *alcohol* (br623_; dummy variables), *country group* (country; dummy variables), *sleep* (mh007; dummy variables). *country_group1*: Latvia, Romania, Lithuania; *country_group2*: Cyprus, Estonia, Hungary, Germany; *country_group3*: Croatia, Bulgaria, Netherlands. ADL, activities of daily living.

### 3.4 Generalized estimating equations

One of our objectives was to evaluate the predictive capacity of SRH for future QoL. Therefore, we first analyzed the distribution of the variable QoL and found a mean of 35.79, a median of 36.0, a SD of 6.424, a skewness of −0.332, and a kurtosis of −0.39. The minimum value was 12, and the maximum value was 48. The 25th percentile was 31, the 50th percentile was 36, and the 75th percentile was 41. We included the same independent variables as those in the linear regression model. For this analysis, we utilized a sample of 3031 participants with a history of diabetes or high blood sugar, who took part in the SHARE waves 7 and 8. The GEE analyses reveal significant model effects (Wald chi‐square test) for SRH (*χ* ^*2*^ = 136,743, *df* = 1, *p* < .05) as well as for the other predictors except for *sex*, number of *chronic diseases*, *polypharmacy*, *pain*, *country groups 2* and *5*, and the interaction between *sex* and SRH. An overview of the model effects is shown in Table 4. The model estimates a significant relationship between QoL and SRH, with a regression coefficient of *β* = −1.261 (Wald chi‐square test, *χ* ^*2*^ = 22,097, *df* = 1, *p* < .05). The beta coefficients of the other variables in the model can be found in supplement table 3.

**TABLE 4:** Predictors of future QoL: GEE tests of model effects.

	Wald chi‐square	df	Sig.
Constant	2075.732	1	0.000
Employment status	16.099	5	0.007
Sex	0.099	1	0.753
Pain	3.643	1	0.056
Physical inactivity	99.707	3	0.000
Polypharmacy	0.299	1	0.584
Hospital	39.391	2	<0.001
Self‐rated health	136.743	1	0.000
Memory	22.097	1	<0.001
Adl	10.724	1	0.001
Chronic diseases	2.939	1	0.086
Depression	537.600	1	0.000
Eyesight	57.094	1	<0.001
Country_group1	18.224	1	<0.001
Country_group2	2.374	1	0.123
Country_group3	22.311	1	<0.001
Country_group4	8.633	1	0.003
Country_group5	0.241	1	0.624
Country_group6	29.209	1	<0.001
Country_group7	22.776	1	<0.001
Sex * self‐rated health	0.713	1	0.398

*Note*: Dependent variable: *CASP index for quality of life and well‐being* ^42^; *country_group1*: Latvia, Romania, Lithuania; *country_group2*: Cyprus, Estonia, Hungary, Germany; *country_group3*: Croatia, Bulgaria, Netherlands; *country_group4*: Slovenia, Slovakia, Sweden; *country_group5*: Malta, Israel, Italy, Spain; *country_group6*: Switzerland, Greece, Austria; *country_group7*: Denmark, Finland, Luxembourg. ADL, activities of daily living; GEE, generalized estimating equations; QoL, quality of life.

## 4 DISCUSSION

SRH is an economical way of measuring health and is often used in large‐scale, population‐based studies, such as SHARE. It summarizes a combination of health‐related and non‐health‐related factors individuals use to rate their health. Chronic conditions, such as diabetes, influence how people rate their health. Therefore it is important to examine the determinants of SRH in people with diabetes.

The aim of this study was to improve the understanding of factors affecting SRH in individuals with diabetes and to evaluate the predictive potential of SRH on QoL. We examined sociodemographic, socioeconomic, medical, social, mental, health care use, and health behavior indicators to determine their contribution to the variance in SRH. We also analyzed the impact of sociodemographic factors and discovered that the respondent's country played a role. In addition to identifying the health information that individuals rely on to make judgments, we also gained insight into the non‐health‐related determinants of SRH in people with diabetes.

Individuals with diabetes more commonly reported a SRH of “less than very good” compared to those without diabetes. Our research identified several factors that significantly affect SRH in individuals with diabetes, including country of residence, polypharmacy, being troubled with pain, memory function, visual problems, limitations with ADL, being affected by chronic diseases, and depression. Together, these factors account for 38% of the variance in SRH in individuals with diabetes. It is worth noting that the variables depression, pain, and memory function have the strongest influence in determining SRH in individuals with diabetes. Thus, when individuals with diabetes evaluate their health, the analyzed variables that exert the most impact on their responses are depressive symptoms, pain experience, and memory function. In other words, these are the key factors influencing their subjective ratings.

*Depression* is the most important determinant of SRH, which is in line with the current research, indicating a firm association between depression and SRH. This finding holds particular importance for individuals with diabetes, as they exhibit heightened likelihood of depressive symptoms as compared to the general population.^68^ Furthermore, depression can affect how individuals with diabetes perceive their illness and symptoms, potentially leading to a greater perceived symptom burden, lack of control, and worse anticipated consequences.^69^ Moreover, individuals with diabetes and depression are more likely to consider their illness to be more severe than those without depression.^70^

Another important factor contributing to the variance of SRH is *pain*. Pain is closely linked to an individual's objective health status, because many diseases cause pain as a symptom. In addition, pain can manifest as a disease in its own right.^71^ Those suffering from diabetes are particularly at risk of experiencing pain, with around half of them developing polyneuropathy, and 25% developing neuropathic pain.^72^, ^73^

Our study discovered that *memory* is the third most influential determinant for explaining the variance in SRH. This is consistent with prior research that has linked SRH with cognitive performance,^74^, ^75^ cognitive impairment,^76^ and dementia.^77^ However, further investigation is needed to ascertain the underlying causes of the association between memory and SRH. It is possible that changes in cognitive abilities are represented in SRH or that other mechanisms are contributing to the decline in both SRH and memory.^78^

We can conclude that the country of the respondent should not be omitted as some of the country dummy variables remained in our model. This influence has various possible reasons that require discussion. The most apparent explanation is that SRH reflects actual health status differences between countries, such as socioeconomic disparities. However, cultural differences may result in varying degrees of health knowledge, response patterns, and differential item functioning.^79^, ^80^ Some individuals, for example, may aspire to present themselves in either a positive or negative light. Also, individuals may rely on different indicators or give different weights to them in their ratings. There may also be linguistic differences due to translation. Although we corrected for age in our model, it is worth to note that the average age per country in our sample ranges from 63.3 years (Slovakia) to 73.4 years (Spain), which may affect SRH.

We found a significant relationship between SRH and QoL after 1 year. Our findings indicate that SRH is an independent predictor of QoL in individuals with diabetes, while controlling for other variables potentially influencing the effect (*chronic diseases*, *eyesight*, *adl*, *depression*, *memory*, *employment status*, *physical inactivity*, *polypharmacy*, *pain*, *hospital*, *country*, and *sex*). This means that participants with diabetes who reported lower SRH during the seventh SHARE wave had lower QoL during the eight wave, whereas those with a higher SRH during the seventh wave had a better QoL during the eight wave. Improving QoL refers to the comprehensive health approach promoted by the World Health Organization.^32^ To improve QoL, it is essential to identify its predictors. This study determines the predictive value of SRH, emphasizing the significance of understanding how SRH ratings are composed.

SRH has been widely studied in epidemiological research but rarely implemented in clinical practice. The clinical use of SRH, such as in routine medical assessments, has been recommended in several publications.^16^, ^29^, ^81^, ^82^, ^83^ As an example, Waller^82^ (p. 2) states: “To disregard the patient's subjective view of health is equal to not using the most valuable piece of information there is”. Despite the significance of practical implementation, limited research has investigated its effects. Waller et al^84^ conducted a qualitative study wherein general practitioners asked their patients in a consultation to rate their health status. In 30 of the 33 consultations, clinicians reported that the question had an impact on their understanding of the patients' health conditions or the consultation itself. Also, the intervention let to an overall increase in patient speaking time during consultations. Several patients responded emotionally, providing valuable insights to the general practitioner regarding their overall situation. The physicians also reported that posing the question changed the consultation atmosphere and created an opportunity for sensitive discussions on matters such as lifestyle, challenging live circumstances, and resources.^84^

SRH offers a simple and efficient method for obtaining a comprehensive understanding of an individual's health trajectory, and focusing on the patient's subjective experience of their own health.^16^ The predictive value of SRH for QoL underlines the importance of recognizing SRH as an essential element in assessing overall health. It can be assumed that enhancements to people's health and their perception of it, may lead to improvements in their future QoL. Considering the widespread incidence of diabetes worldwide, our findings hold great significance for the affected population. Health care professionals may aid these patients in obtaining better health outcomes and a higher QoL by addressing all variables contributing to SRH in people with diabetes.

This study is the first to analyze determinants of SRH and its relationship to future QoL in people with diabetes, though it has some limitations. The use of SHARE limits the generalizability of the results, as a possible selection bias cannot be excluded. It is likely that individuals in nursing homes or hospitals or those who are incapacitated to participate in interviews due to cognitive or functional impairments are underrepresented in this dataset. Additionally, the distinction between Type I and Type II diabetes was not considered in the dataset, and only the overall diagnosis of diabetes was assessed. Although 38% of the variance in our model can be attributed to the analyzed SHARE data, more than half of the variance remains unexplained. In addition to the factors identified in our study, SRH in people with diabetes may be influenced by factors that are not covered in our research. For example, health knowledge, respondent or interviewer characteristics, and bias might play a significant role. Future research should delve into these aspects to identify additional sources of variance and gain a more comprehensive understanding of the determinants of SRH in people with diabetes.

## AUTHOR CONTRIBUTIONS

Tino Prell: Design, statistical analyses, drafting the work. Rosa Marie Brückner: statistical analyses, interpretation of data, revising it critically for important intellectual content. Rebecca Wientzek: interpretation of data and revising it critically for important intellectual content. Aline Schönenberg: interpretation of data and revising it critically for important intellectual content. Mandy Schreiber: statistical analyses, revising manuscript critically for important intellectual content. All authors reviewed the final manuscript and agreed to be accountable for all aspects of the work in ensuring that questions related to the accuracy or integrity of any part of the work are appropriately investigated and resolved.

## FUNDING INFORMATION

Tino Prell received funding from the Bundesministerium für Bildung und Forschung (Federal Ministry of Education and Research) grant (01GY2301).

## DISCLOSURE

The authors report no conflict of interests.

## Supporting information


**TABLE S1.** Countries with similar influence on SRH grouped according to their standardized coefficients beta. SRH, self‐rated health.
**TABLE S2.** Characteristics of people with and without diabetes or high blood sugar.
**TABLE S3.** Estimates of regression parameters.

## Data Availability

This paper uses data from SHARE Waves 7 and 8 (DOIs: 10.6103/SHARE.w7.800, 10.6103/SHARE.w8.800) see Börsch‐Supan et al^14^ for methodological details. The SHARE data collection has been funded by the European Commission, DG RTD through FP5 (QLK6‐CT‐2001‐00360), FP6 (SHARE‐I3: RII‐CT‐2006‐062193, COMPARE: CIT5‐CT‐2005‐028857, SHARELIFE: CIT4‐CT‐2006‐028812), FP7 (SHARE‐PREP: GA N°211 909, SHARE‐LEAP: GA N°227 822, SHARE M4: GA N°261 982, DASISH: GA N°283 646) and Horizon 2020 (SHARE‐DEV3: GA N°676 536, SHARE‐COHESION: GA N°870 628, SERISS: GA N°654 221, SSHOC: GA N°823 782, SHARE‐COVID19: GA N°101 015 924) and by DG Employment, Social Affairs & Inclusion through VS 2015/0195, VS 2016/0135, VS 2018/0285, VS 2019/0332, and VS 2020/0313. Additional funding from the German Ministry of Education and Research, the Max Planck Society for the Advancement of Science, the U.S. National Institute on Aging (U01_AG09740‐13S2, P01_AG005842, P01_AG08291, P30_AG12815, R21_AG025169, Y1‐AG‐4553‐01, IAG_BSR06‐11, OGHA_04–064, HHSN271201300071C, RAG052527A) and from various national funding sources is gratefully acknowledged (see www.share-project.org). Data are freely available for scientific use after an initial registration at the SHARE homepage.
