# Supplementary material for: Exploring factors associated with self‐rated health in individuals with diabetes and its impact on quality of life: Evidence from the Survey of Health, Ageing, and Retirement in Europe
Source: J Diabetes. 2024 Jan 2;16(8):e13522. doi: 10.1111/1753-0407.13522 (PMC11333853; doi:10.1111/1753-0407.13522)
Supplement: Supplementary file 1 — TABLE S1. Countries with similar influence on SRH grouped according to their standardized coefficients beta. SRH, self‐rated health. TABLE S2. Characteristics of people with and without diabetes or high blood sugar. TABLE S3. Estimates of regression parameters. [file JDB-16-e13522-s001.pdf]

Supporting Information

Supplement table 1. Countries with similar influence on SRH grouped according to their standardized coefficients beta

Country group	Country	Standardized beta
country_group1	Latvia	0.130
	Romania	0.040
	Lithuania	0.031
country_group2	Cyprus	0.020
	Estonia	0.019
	Hungary	0.007
	Germany	0.004
country_group3	Croatia	-0.002
	Bulgaria	-0.005
	Netherlands	-0.010
country_group4	Slovenia	-0.013
	Slovakia	-0.014
	Sweden	-0.015
country_group5	Malta	-0.026
	Israel	-0.032
	Italy	-0.037
	Spain	-0.037
country_group6	Switzerland	-0.050
	Greece	-0.050
	Austria	-0.051
country_group7	Denmark	-0.069
	Finland	-0.076
	Luxembourg	-0.125

Note. Linear model; dependent variable: self-rated health; independent variables:, country=Luxembourg, country=Sweden, country=Croatia, country=Italy, country=Latvia, country=Hungary, country=Switzerland, country=Israel, country=Slovenia, country=Germany, country=Malta, country=Bulgaria, country=Greece, country=Netherlands, country=Estonia, country=Spain, country=Austria, country=Cyprus, country=Slovakia, country=Romania, country=Lithuania, country=Denmark, country=Finland, employment status=Other, employment status=Homemaker, employment status=Unemployed, employment status=Permanently sick or disabled, employment status= Employed or self-employed (including working for family business), marital status=Married, not living with spouse, marital status=Divorced, marital status=Widowed, marital status=Registered partnership, marital status=Never married, loneliness, hospital=Yes, physical inactivity=Yes, smoke=No, sn satisfaction, chronic diseases, Household net worth, age diabetes, education, Body mass index, sn size, hearing, adl, alcohol=Three or four days a week, alcohol=Not at all in the last 3 months, alcohol=Daily or almost daily, alcohol=Once or twice a week, alcohol=Five or six days a week, alcohol=Once or twice a month, pain=Yes, fluency, eyesight, gender=Female, health literacy, polypharmacy_=No, memory, depression, age, grip strength, sn scale

Supplement table 2. Characteristics of people with and without diabetes or high blood sugar

	Never had diabetes or high blood sugar (N=39668)	Ever had/currently have diabetes or high blood sugar (N=6924)	Total (N=46592)	p-value (Chi-Square Test or T-test)	Cohen´s d or Cramers V
**self rated health**				<.001	-0.536
Excellent	2536 (6.4%)	82 (1.2%)	2618 (5.6%)		
Very good	7148 (18.0%)	455 (6.6%)	7603 (16.3%)		
Good	15468 (39.0%)	2283 (33.0%)	17751 (38.1%)		
Fair	10872 (27.4%)	2817 (40.7%)	13689 (29.4%)		
Poor	3617 (9.1%)	1284 (18.6%)	4901 (10.5%)		
**self-rated health, dichotomized**				<.001	0.143
Very good/excellent	9684 (24.4%)	537 (7.8%)	10221 (22.0%)		
Less than very good	29957 (75.6%)	6384 (92.2%)	36341 (78.0%)		
**age**				<.001	-0.269
Mean (SD)	69.8 (9.6)	72.4 (8.6)	70.2 (9.5)		
**gender**				<.001	0.044
Male	16494 (41.6%)	3306 (47.7%)	19800 (42.5%)		
Female	23174 (58.4%)	3618 (52.3%)	26792 (57.5%)		
**marital status**					
Married, living with spouse	26457 (66.7%)	4372 (63.1%)	30829 (66.2%)	<.001	0.053
Registered partnership	579 (1.5%)	66 (1.0%)	645 (1.4%)		
Married, not living with spouse	395 (1.0%)	81 (1.2%)	476 (1.0%)		
Never married	1983 (5.0%)	335 (4.8%)	2318 (5.0%)		
Divorced	3336 (8.4%)	491 (7.1%)	3827 (8.2%)		
Widowed	6918 (17.4%)	1579 (22.8%)	8497 (18.2%)		
**country**				<.001	0.115
Austria	1341 (3.4%)	226 (3.3%)	1567 (3.4%)		
Germany	2423 (6.1%)	455 (6.6%)	2878 (6.2%)		
Sweden	2071 (5.2%)	283 (4.1%)	2354 (5.1%)		
Netherlands	1721 (4.3%)	211 (3.0%)	1932 (4.1%)		
Spain	1712 (4.3%)	403 (5.8%)	2115 (4.5%)		
Italy	1872 (4.7%)	295 (4.3%)	2167 (4.7%)		
France	2164 (5.5%)	311 (4.5%)	2475 (5.3%)		
Denmark	1974 (5.0%)	194 (2.8%)	2168 (4.7%)		
Greece	2514 (6.3%)	484 (7.0%)	2998 (6.4%)		
Switzerland	1733 (4.4%)	169 (2.4%)	1902 (4.1%)		
Belgium	1748 (4.4%)	252 (3.6%)	2000 (4.3%)		
Israel	676 (1.7%)	245 (3.5%)	921 (2.0%)		
Czech Republic	2131 (5.4%)	580 (8.4%)	2711 (5.8%)		
Poland	1652 (4.2%)	423 (6.1%)	2075 (4.5%)		
Luxembourg	832 (2.1%)	121 (1.7%)	953 (2.0%)		
Hungary	591 (1.5%)	182 (2.6%)	773 (1.7%)		
Slovenia	2084 (5.3%)	412 (6.0%)	2496 (5.4%)		
Estonia	2588 (6.5%)	431 (6.2%)	3019 (6.5%)		
Croatia	1008 (2.5%)	180 (2.6%)	1188 (2.5%)		
Lithuania	1287 (3.2%)	148 (2.1%)	1435 (3.1%)		
Bulgaria	771 (1.9%)	131 (1.9%)	902 (1.9%)		
Cyprus	417 (1.1%)	119 (1.7%)	536 (1.2%)		
Finland	984 (2.5%)	176 (2.5%)	1160 (2.5%)		
Latvia	712 (1.8%)	76 (1.1%)	788 (1.7%)		
Malta	643 (1.6%)	161 (2.3%)	804 (1.7%)		
Romania	1114 (2.8%)	166 (2.4%)	1280 (2.7%)		
Slovakia	905 (2.3%)	90 (1.3%)	995 (2.1%)		
**education**				<.001	0.217
Mean (SD)	11.4 (4.2)	10.5 (4.1)	11.2 (4.2)		
**health literacy**				<.001	0.238
Always	2341 (5.9%)	674 (9.8%)	3015 (6.5%)		
Often	1324 (3.4%)	370 (5.4%)	1694 (3.7%)		
Sometimes	3114 (7.9%)	712 (10.3%)	3826 (8.2%)		
Rarely	3922 (9.9%)	798 (11.6%)	4720 (10.2%)		
Never	28790 (72.9%)	4340 (63.0%)	33130 (71.4%)		
**net worth**				<.001	0.067
Mean (SD)	305207 (1582410.1)	207019.3 (483356.1)	290615.4 (1472354.8)		
**employment status**				<.001	0.111
Retired	26421 (67.4%)	5289 (77.6%)	31710 (68.9%)		
Employed or self-employed (including working for family business)	7775 (19.8%)	578 (8.5%)	8353 (18.2%)		

Unemployed	721 (1.8%)	93 (1.4%)	814 (1.8%)		
Permanently sick or disabled	922 (2.4%)	267 (3.9%)	1189 (2.6%)		
Homemaker	2805 (7.2%)	489 (7.2%)	3294 (7.2%)		
Other	562 (1.4%)	100 (1.5%)	662 (1.4%)		
**bmi**				<.001	-0.546
Mean (SD)	26.8 (4.6)	29.3 (5.3)	27.2 (4.8)		
**chronic diseases**				<.001	-1.204
Mean (SD)	1.7 (1.5)	3.5 (1.7)	1.9 (1.6)		
**polypharmacy**				<.001	0.266
Yes	7897 (26.0%)	3972 (58.0%)	11869 (31.9%)		
No	22459 (74.0%)	2876 (42.0%)	25335 (68.1%)		
**physical inactivity**				<.001	0.097
No	34883 (87.9%)	5444 (78.6%)	40327 (86.6%)		
Yes	4785 (12.1%)	1480 (21.4%)	6265 (13.4%)		
**grip strength**					
Mean (SD)	32.2 (11.2)	30.7 (11.1)	32.0 (11.2)		
**eyesight**				<.001	0.076
Excellent	6236 (15.7%)	797 (11.5%)	7033 (15.1%)		
Very good	11496 (29.0%)	1703 (24.6%)	13199 (28.3%)		
Good	15463 (39.0%)	2855 (41.2%)	18318 (39.3%)		
Fair	4633 (11.7%)	1051 (15.2%)	5684 (12.2%)		
Poor	1840 (4.6%)	518 (7.5%)	2358 (5.1%)		
**hearing**				<.001	0.077
Excellent	5619 (14.2%)	670 (9.7%)	6289 (13.5%)		
Very good	10449 (26.3%)	1516 (21.9%)	11965 (25.7%)		
Good	15994 (40.3%)	2978 (43.0%)	18972 (40.7%)		
Fair	6301 (15.9%)	1390 (20.1%)	7691 (16.5%)		
Poor	1305 (3.3%)	370 (5.3%)	1675 (3.6%)		
**pain**					
Yes	17280 (43.6%)	3773 (54.6%)	21053 (45.3%)		
No	22335 (56.4%)	3135 (45.4%)	25470 (54.7%)		
**adl**					-0.231
Mean (SD)	0.3 (0.9)	0.5 (1.2)	0.3 (1.0)		
**sn_size**					
Mean (SD)	2.7 (1.6)	2.5 (1.6)	2.7 (1.6)		
**sn_satisfaction**				0.122	0.021
Mean (SD)	8.9 (1.4)	8.9 (1.5)	8.9 (1.4)		
**sn_scale**				<.001	0.089
Mean (SD)	1.9 (1.0)	1.8 (1.0)	1.9 (1.0)		
**loneliness**				<.001	-0.189
Mean (SD)	3.9 (1.4)	4.2 (1.6)	4.0 (1.4)		
**depression**				<.001	-0.231
Mean (SD)	2.4 (2.2)	2.9 (2.4)	2.4 (2.3)		
**fluency**				<.001	0.270
Mean (SD)	20.4 (7.8)	18.3 (7.3)	20.1 (7.8)		
**memory**				<.001	-0.185
Excellent	2539 (6.4%)	340 (4.9%)	2879 (6.2%)		
Very good	8162 (20.6%)	1085 (15.7%)	9247 (19.8%)		
Good	18242 (46.0%)	3164 (45.7%)	21406 (45.9%)		
Fair	9045 (22.8%)	1865 (26.9%)	10910 (23.4%)		
Poor	1680 (4.2%)	470 (6.8%)	2150 (4.6%)		
**sleep**				<.001	0.040
Trouble with sleep or recent change in pattern	13989 (35.9%)	2783 (41.3%)	16772 (36.7%)		
No trouble sleeping	24998 (64.1%)	3960 (58.7%)	28958 (63.3%)		
**hospital**				<.001	0.068
No	33794 (85.2%)	5414 (78.2%)	39208 (84.2%)		
Yes	5874 (14.8%)	1510 (21.8%)	7384 (15.8%)		
**nursing home**					
Yes, temporarily	127 (0.3%)	30 (0.4%)	157 (0.3%)		
Yes, permanently	11 (0.0%)	5 (0.1%)	16 (0.0%)		
No	39070 (99.6%)	6776 (99.5%)	45846 (99.6%)		
**smoke**				<.001	0.046
Yes	5684 (35.1%)	844 (29.0%)	6528 (34.2%)		
No	10495 (64.9%)	2065 (71.0%)	12560 (65.8%)		
**alcohol**				<.001	0.049
Daily or almost daily	679 (1.7%)	84 (1.2%)	763 (1.6%)		
Five or six days a week	253 (0.6%)	29 (0.4%)	282 (0.6%)		
Three or four days a week	553 (1.4%)	63 (0.9%)	616 (1.3%)		
Once or twice a week	1316 (3.3%)	170 (2.5%)	1486 (3.2%)		
Once or twice a month	2096 (5.3%)	272 (3.9%)	2368 (5.1%)		
Less than once a month	3519 (8.9%)	473 (6.8%)	3992 (8.6%)		
Not at all in the last 3 months	31177 (78.7%)	5823 (84.2%)	37000 (79.6%)		

Supplement table 3. Estimates of regression parameters

	Beta	Std.-Error	95% Wald Confidence Interval Lower	Upper	Wald-Chi-Square Test	df	Sig.
**(constant)**	42.645	1.4888	39.727	45.563	820.479	1	.000
**Self-rated health**	-1.261	.1528	-1.561	-.962	68.162	1	<.001
**Memory**	-.448	.0954	-.635	-.261	22.097	1	<.001
**adl**	-.292	.0890	-.466	-.117	10.724	1	.001
**Chronic diseases**	-.098	.0571	-.210	.014	2.939	1	.086
**Depression**	-.958	.0413	-1.039	-.877	537.600	1	.000
**Eyesight**	-.623	.0825	-.785	-.462	57.094	1	<.001
**Current job situation**							
Retired	.475	.6514	-.802	1.752	.532	1	.466
Employed or self-employed (including working for family business)	.987	.6976	-.381	2.354	2.000	1	.157
Unemployed	-.724	.8974	-2.483	1.035	.652	1	.420
Permanently sick or disabled	-.250	.7698	-1.759	1.259	.106	1	.745
Homemaker	-.454	.7250	-1.874	.967	.391	1	.532
Other	0a	.	.	.	.	.	.
**Sex**							
Male	.210	.6671	-1.097	1.518	.099	1	.753
Female	0a	.	.	.	.	.	.
**Pain**							
Yes	-.326	.1706	-.660	.009	3.643	1	.056
No	0a	.	.	.	.	.	.
**Physical inactivity**							
Refusal	4.269	2.9658	-1.544	10.082	2.072	1	.150
Don’t know	6.034	.7675	4.529	7.538	61.803	1	<.001
Other	1.869	.2341	1.410	2.327	63.730	1	<.001
Never vigorous nor moderate physical activity	0a	.	.	.	.	.	.
**Country**							
Country_group2=0	-.371	.2409	-.843	.101	2.374	1	.123
Country_group2=1	0a	.	.	.	.	.	.
Country_group4=0	-.772	.2628	-1.287	-.257	8.633	1	.003
Country_group4=1	0a	.	.	.	.	.	.
Country_group5=0	-.126	.2577	-.631	.379	.241	1	.624
Country_group5=1	0a	.	.	.	.	.	.
Country_group7=0	-1.408	.2950	-1.986	-.830	22.776	1	<.001
Country_group7=1	0a	.	.	.	.	.	.
Country_group1=0	1.686	.3950	.912	2.460	18.224	1	<.001
Country_group1=1	0a	.	.	.	.	.	.
Country_group3=0	1.824	.3863	1.067	2.582	22.311	1	<.001
Country_group3=1	0a	.	.	.	.	.	.
Country_group6=0	1.743	.3226	1.111	2.376	29.209	1	<.001
Country_group6=1	0a	.	.	.	.	.	.
**polypharmacy**							
Yes	-.096	.1752	-.439	.248	.299	1	.584
No	0a	.	.	.	.	.	.
**hospital**							
Don’t know	4.406	.7328	2.970	5.842	36.153	1	<.001
Yes	.487	.1940	.107	.868	6.305	1	.012
No	0a	.	.	.	.	.	.
**Interaction sex*self-rated health**							
Sex = male * Self-rated health	-.152	.1805	-.506	.201	.713	1	.398
Sex = female * Self-rated health	0a	.	.	.	.	.	.
scale	24.035						

Note. Dependent variable: CASP index for quality of life and well-being (Higgs et al., 2003); a. set to 0, since parameter is redundant.
